# Snake diversity, occupancy, and detection on Thailand's largest university campus

**DOI:** 10.1002/ece3.70317

**Published:** 2024-09-18

**Authors:** Curt H. Barnes, Ungku Zafirah Abdulaziz, Arwut Kaenphet, Chatchai Kanlayanapaphon

**Affiliations:** ^1^ Walailak University Nakhon Si Thammarat Thailand; ^2^ Universiti Sains Malaysia, USM Penang Malaysia

**Keywords:** Bayesian occupancy analysis, detection probability, diversity, snake, Walailak university

## Abstract

More than 240 species of snake have been described from Thailand, yet basic natural history and ecology for this group of animals remains scarce in human disturbed environments despite conservation and human medical significance of them in these habitats. We studied snake diversity at Walailak University from March to December 2023, the largest university campus in Thailand (1525 hectares) through standardized walking surveys, opportunistic notifications and observation, road surveys, and traps and evaluated diversity using the Shannon diversity index (*H*), Pielou's evenness of species (*J*), detection probabilities (*p*), and occupancy probabilities (ψ). We observed 195 snakes (21 species, 7 families) at Walailak University and overall snake diversity (*H* = 2.60) and evenness (*J* = 0.85) were quite high, although specific site diversity (range *H* = 0–1.94) and evenness (range *J* = 0.67–0.91) within the university were variable. The probability of detecting snakes (range *p* = .10–.40) increased with increasing humidity and decreased with increasing amount of rain, temperature, and wind; site occupancy probability decreased with increased canopy height and increased with increased distance to buildings, increased canopy height loss, increased distance to roads, and increased distance to water. Our findings of relatively high snake diversity, presence of snake species potentially dangerous to humans (six species), and protected snake species (Thailand WARPA and international CITES, five species) suggest significant potential for conservation and further research at Walailak University and other campuses in Thailand.

## Graphical abstract

More than 240 species of snake have been described from Thailand, yet basic natural history and ecology for this group of animals remains scarce in human disturbed environments despite conservation and human medical significance of them in these habitats. We studied snake diversity at Walailak University from March to December 2023, the largest university campus in Thailand (1525 hectares) through standardized walking surveys, opportunistic notifications and observation, road surveys, and traps and evaluated diversity using the Shannon diversity index (*H*), Pielou's evenness of species (*J*), detection probabilities (*p*), and occupancy probabilities (ψ). Our findings of relatively high snake diversity, presence of snake species potentially dangerous to humans (six species), and protected snake species (Thailand WARPA and international CITES, five species) suggest significant potential for conservation and further research at Walailak University and other campuses in Thailand.

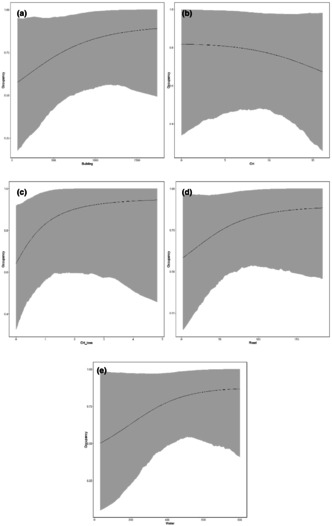

## สรุป

ในประเทศไทยพบงูมากกว่า 240 ชนิด แต่ข้อมูลพื้นฐานเกี่ยวกับธรรมชาติวิทยาและนิเวศวิทยาของงูในสภาพแวดล้อมที่มีการรบกวนจากมนุษย์ยังคงมีน้อย แม้จะมีความสำคัญต่อการอนุรักษ์และการแพทย์ในถิ่นที่อยู่อาศัยเหล่านี้ก็ตาม ทำการศึกษาความหลากหลายของงูที่มหาวิทยาลัยวลัยลักษณ์ซึ่งเป็นมหาวิทยาลัยที่มีพื้นที่ใหญ่ที่สุดในประเทศไทย (1525 เฮกตาร์) ตั้งแต่เดือนมีนาคมถึงเดือนธันวาคม 2566 โดยใช้การเดินเท้าสำรวจ การรายงานเหตุการณ์หรือสิ่งที่สังเกตเจอ แบบไม่ได้มีการวางแผนเก็บข้อมูลตามปกติการสำรวจตามถนน และการใช้กับดัก ทำการประเมินความหลากหลายโดยใช้ดัชนีความหลากหลายของ แชนนอน‐ไวเนอร์ (*H*), ดัชนีความสม่ำเสมอทางชนิดพันธุ์ของพีลู (*J*), ความน่าจะเป็นในการสำรวจพบงู (*p*), และความน่าจะเป็นของการครอบครองเชิงพื้นที่ (ψ) พบงูจำนวน 195 ตัว (21 ชนิด, 7 วงศ์) และพบว่าความหลากหลายของงู (*H* = 2.60) และความสม่ำเสมอ (*J* = 0.85) โดยรวมค่อนข้างสูง แม้ว่าความหลากหลาย (*H* = 0–1.94) และความสม่ำเสมอ (*J* = 0.67–0.91) แต่ละพื้นที่ภายในมหาวิทยาลัยจะแตกต่างกันไป ความน่าจะเป็นในการสำรวจพบงู (*p* = .10–.40) เพิ่มขึ้นตามความชื้นที่เพิ่มขึ้น และลดลงเมื่อปริมาณฝน อุณหภูมิ และลมเพิ่มขึ้น; ความน่าจะเป็นของการครอบครองเชิงพื้นที่ลดลงเมื่อความสูงของเรือนยอดเพิ่มขึ้น และเพิ่มขึ้นเมื่อระยะห่างจากอาคารเพิ่มขึ้น การสูญเสียชั้นเรือนยอดเพิ่มขึ้น ระยะห่างจากถนนเพิ่มขึ้น และระยะห่างจากแหล่งน้ำเพิ่มขึ้น จากการศึกษาพบว่ามีความหลากหลายของงูค่อนข้างสูง มีชนิดของงูที่อาจเป็นอันตรายต่อมนุษย์ (6 ชนิด) และชนิดงูที่เป็นสัตว์ป่าคุ้มครอง (พระราชบัญญัติสงวนและคุ้มครองสัตว์ป่า และ อนุสัญญาไซเตส, 5 ชนิด) แสดงให้เห็นถึงศักยภาพที่สำคัญสำหรับการอนุรักษ์และการวิจัยเพิ่มเติมที่มหาวิทยาลัยวลัยลักษณ์และมหาวิทยาลัยอื่น ๆ ในประเทศไทย

## 1 INTRODUCTION

More than 4100 snake species have been described world‐wide (Pincheira‐Donoso et al., 2013; Uetz et al., 2024), and this diverse vertebrate group serve as both predators and prey within ecosystems. Yet, approximately 19% of snake species are threatened with extinction (Böhm et al., 2013). Additionally, it has been estimated that more than 1 million people globally are bitten by medically significant species; species which can produce debilitating wounds or even mortality (Ralph et al., 2022) which produce negative perceptions towards this taxon. Studies of snakes in areas of human disturbance are much scarcer than in more pristine and protected areas. Investigation of how these organisms persist in environments alongside humans has received limited attention, despite their role as predators and prey in the ecology of these habitats, decline of many species, and potential for dangerous interactions with humans. Universities in Thailand possess high potential for mitigating this knowledge gap as campuses typically value green spaces (for example, retention of trees and shrubs and creation of ponds) for human esthetics. These values may also align with snake basic ecological needs and there is interest at these places in promotion of science, including biology, thus providing potential opportunities for inclusion of students, faculty, staff, and general public. We provide the first comprehensive study devoted to snake diversity on a university campus in Thailand, Walailak University, which is also the largest in the country by area.

We anticipated that snake species diversity would be higher at Walailak University than smaller universities in Thailand (five species minimum observed), with at least 10 species observed. We present the first occupancy and detection probability analyses results for multiple snake species at any site in Thailand, but we predict that for site occupancy variables (1) snakes will be observed further away from anthropogenic disturbances (buildings and roads), (2) be observed closer to water, (3) will be observed in areas with higher canopy cover, and (4) be observed in areas with less canopy cover loss; for survey detection variables, we predict (1) snakes will be more likely to be observed in warmer temperatures, (2) will be more likely to be observed during higher humidity, (3) will be more likely to be observed during higher precipitation, and (4) will be less likely to be observed during high wind speeds.

## 2 MATERIALS AND METHODS

### 2.1 Study area

**FIGURE 1:**
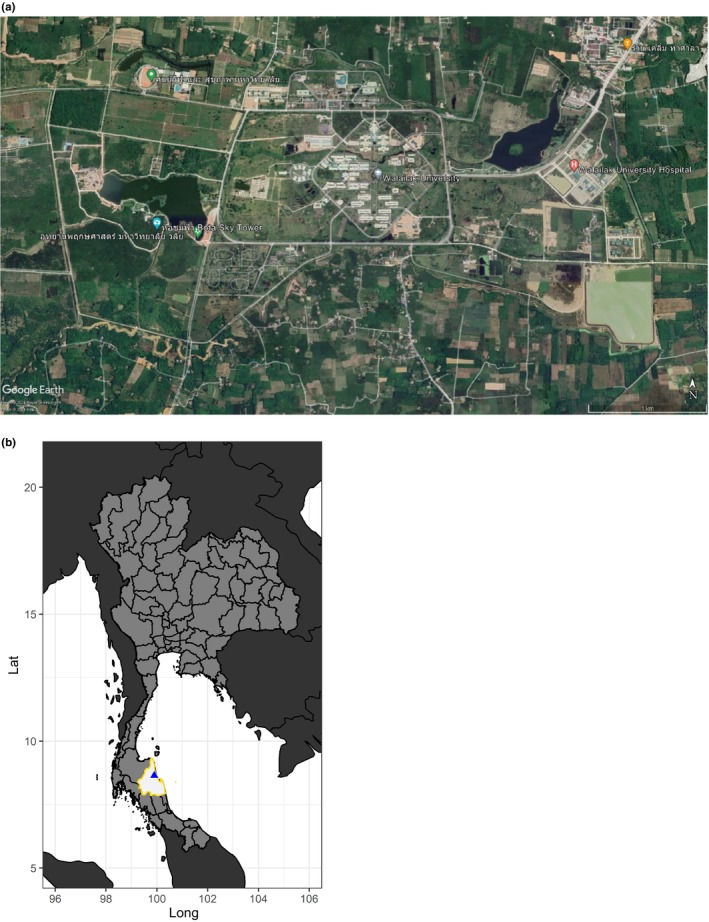
Study site (a) Walailak University, located in Nakhon Si Thammarat province in the southern part of Thailand, derived from Google Earth, with (b) location within the country provided (Thailand as light gray, Nakhon Si Thammarat in white outlined in yellow, and Walailak University as a blue triangle). University area is approximately 1525 hectares.

Founded in 1992, Walailak University is the largest university campus by area in Thailand, comprised of over 1525 hectares (Figure 1). Located in southern Thailand within Nakhon Si Thammarat province, many land use types are present on the campus including university buildings (such as hospital, student and staff dormitories, lecture halls, and laboratories), ponds, streams, forest patches (about 375 hectares), and a large park (40 hectares) and a botanical garden (216 hectares). Rubber (*Hevea brasiliensis*), durian (*Durio zibethinus*), and oil palm (*Elaeis guineensis*) plantations are present on campus. Cattle of multiple varieties are grazed throughout the campus, also. There were around 10,000 students, staff, and faculty at Walailak University during the study period.

### 2.2 Data collection

We attempted to conduct each survey opportunistically walking for an hour, although some sites did take longer or shorter depending on environmental or site‐specific characteristics. Standardized surveys were primarily conducted at night (between 1800 and 2300) due to logistical constraints; headlights were used to search for snakes at night. Opportunistic observations (non‐standardized) and traps (two funnel traps at the end of 10 m long drift fence line per array; five arrays total; checked at least once per day) were utilized for general diversity analysis.

### 2.3 Data analysis

#### 2.3.1 Diversity

Species diversity (alpha) was investigated at two scales, (1) Walailak University as a single area, which utilized data from standardized surveys (same data, 20 sites, as occupancy and detection probability analyses, Section 2.3.2 (Occupancy and detection probability)), opportunistic observations, and traps, and (2) sites within Walailak University, which only utilized standardized surveys. Specific site details are not provided due to comparatively small overall study area size which could result in disturbance, collection, or mortality of snakes observed (as discussed and observed in previous studies‐Lindenmayer & Scheele, 2017; Stuart et al., 2006), possible even with masking by journals and partial publication (Lowe et al., 2017). We utilized the “vegan” package (Oksanen et al., 2016) in program R (R Development Core Team, 2018) to investigate diversity. The Shannon index (*H′*; Shannon, 1948) was calculated using the function “diversity.” We sought to understand the influence of sample size, number of snake species observed, on species richness and utilized the function “rarefy” to create rarefaction curves for both diversity scales. For the multisite scale, we also created species accumulation curves using the “specaccum” function to understand the influence of number of sites on species richness. Pielou's evenness (Pielou, 1966) was calculated using program R through the calculation *J* = *H′*/log(*S*), where *J* is evenness, *H*′ is diversity, and *S* is number of species–the Shannon diversity estimate derived from function “diversity” divided by the log of number of species.

#### 2.3.2 Occupancy and detection probability

Bayesian single‐season models were utilized to estimate the occupancy and detection probability of snakes at 20 sites within Walailak University in 2023. Occupancy (ψ) variables (estimated in m) we thought could be important to snake occupancy and subsequently modeled with detection held constant (no variables; p.) included closest distance to water (m), closest distance to houses and buildings (m), closest distance to roads (m), canopy height (m), and canopy height loss (m). Temperature (°C), humidity (%), rain (cm), and wind speed (km/h) were modeled as detection (p) variables with the site held constant (no variables; ψ).

Detection variables, temperature, humidity, rain, and wind speed were derived from the “microclima” R package (Maclean et al., 2019). The closest distance to buildings, roads, and water site variables were estimated using satellite imagery (obtained from the “osm” R package; Padgham et al., 2017) and Euclidean distance (using the “st_distance” function in the “sf” package of R; Pebesma, 2018) in program R. Canopy height was obtained from Dubayah et al. (2021) (1 km. resolution), and canopy height loss was derived from Potapov et al. (2022) (30 m. resolution).

We did not attempt to run all combinations (“dredging”) of variables into models due to the exploratory nature of this study, potential for correlation of variables, and promotion of scientific analysis best practice (particularly avoiding the fallacy “hypotheses after results are known”, HARKING; Kerr, 1998). Due to lack of previous occupancy and detection modeling of general herpetofauna in Thailand, uninformative priors (Logistic (0,1)) were utilized.

All single‐season models (and evaluation of models) were conducted in R with the “ubms” package (Bürkner, 2017), which fits occupancy models in a Bayesian framework using Stan (Carpenter et al., 2017). For each model, we specified three chains run for 25,000 warmup and 50,000 postwarmup iterations, for a minimum total of 55,000 simulation draws. We reviewed Gelman‐Rubin statistic Rhat values and trace plots to check for Markov chain Monte Carlo (MCMC) chain convergence (Gelman & Rubin, 1992) and used MacKenzie–Bailey chi‐square tests (MacKenzie & Bailey, 2004) to assess model goodness‐of‐fit. Models were compared to each other using the leave‐one‐out cross‐validation information criterion (LOOIC; Vehtari et al., 2019). Credible intervals (CRI) are presented alongside parameter estimates (logit scale), which may be interpreted similar to that of a confidence interval but instead provide a range of posterior values that includes 95% of the probability. The inference of the covariate effect was evaluated based on those credible intervals and whether or not they overlapped 0 (strong effect = CRI do not overlap zero, weak effect = CRI overlap zero). Diversity and general model formation are archived on Open Science Framework database (diversity, https://osf.io/qgn2p/?view_only=d15e1f7becfa44e5a106111c6069297b; occupancy and detection, https://osf.io/sb37t/?view_only=874f6b5c08e74e4092b483a27d936af6).

### 2.4 Ethics statement

Research and ethics permissions were approved by Walailak University (WU‐ACUC‐66012) and the365 National Research Council of Thailand (0401/4062), with Thai IACUC Institute of Animals for Scientific366 Purpose Development (IAD) licensure under C.H. Barnes. Our research adhered to Guidelines for367 Research on Live Amphibians and Reptiles (American Herpetological Animal Care and Use Committee; 368 HACC, 2004) ethical standards.

## 3 RESULTS

A total of 195 individual snakes of 21 species of seven families (Table S1) were encountered on Walailak University campus during standardized surveys (five of 20 sites, 204 surveys, 178 surveyor hours, 60 snakes), opportunistically (126 snakes), and in traps (nine captures). Trap effectiveness was low due to heat and presence of ants, frequently requiring checks twice per day; traps were only able to be open for approximately a single week each month which resulted in few captures (1 *Ahaetulla* sp., 2 *Calloselasma rhodostoma*, 1 *Dendrelaphis pictus*, 1 *Hypsiscopus plumbea*, 2 *Rhabdophis siamensis*, 1 *Pareas margaritophorus*, and 1 *Ptyas korros* which was immediately released). The most frequently encountered species included Indochinese ratsnakes (*P. korros*, 36 individuals), plumbeous water snakes (*H. plumbea*, 34 individuals), and Malayan pit vipers (*C. rhodostoma*, 22 individuals). Species only encountered once included Mueller's blind snake (*Argyrophis muelleri*), banded krait (*Bungarus fasciatus*), small‐spotted coral snake (*Calliophis maculiceps*), and Laotian wolf snake (*Lycodon laoensis*).

Species protected locally (Thailand; Wildlife Conservation and Protection Act 2562, WARPA) included the radiated ratsnake (*Coelognathus radiatus*), reticulated python (*Python reticulatus*), *P. korros*, sunbeam snake (*Xenopeltis unicolor*), and internationally protected (global; Convention on International Trade in Endangered Species, CITES, Appendix II) species included monocled cobra (*Naja kaouthia*), and *P. reticulatus*. One species, *P. korros*, has been proposed as “Near‐Threatened” with extinction internationally (IUCN Red List), and none of the species observed in our study have been proposed as threatened with extinction locally (Thai Red Data List); worth pointing out is these evaluations (IUCN Red List and Thai Red Data List) do not by themselves provide protection. Snake species at Walailak University encountered during the study which could potentially inflict severe wounds, bleeding, limb loss, organ failure, or death include the banded krait (*B. fasciatus*), small‐spotted coral snake (*C. maculiceps*), Malayan pit viper (*C. rhodostoma*), monocled cobra (*N. kaouthia*), red‐necked keelback (*R. siamensis*), and the non‐venomous reticulated python (*P. reticulatus*).

### 3.1 Diversity

Overall snake diversity at Walailak University was quite high (*H* = 2.60) and rarefaction appeared to begin to plateau (Figure S1). Individual site diversity within Walailak University was variable (*H* = 0–1.94, Table S2), although rarefaction of none of the sites with observations (five sites) appeared to plateau (Figure S2). Additionally, accumulation did not plateau (Figure S3). Evenness was relatively high for all sites collectively (*J* = 0.85) but varied between sites with detections (range *J* = 0.67–0.91, Table S2).

### 3.2 Occupancy and detection probability

The information criteria (leave‐one‐out; LOO) of the survey detection models temperature, humidity, rain, and wind, and site occupancy models buildings, roads, water, canopy height, and canopy height loss were all highly similar (range 209.45–215.485, Table 1). Of the detection models, wind speed performed best and temperature performed least well. Canopy height loss performed best of the site occupancy models, and canopy height the least well. Fit of all models collectively was centered roughly around 0.5 (range 0.44–0.69).

**TABLE 1:** Summary of single season Bayesian models for estimating snake detection probability and occupancy information criterion (leave‐one‐out; LOO) and model fit (goodness‐of‐fit; GOF) at Walailak University in 2023.

Model type	Model	LOOIC	GOF
Detection	Humidity	214.779	0.44
	Rain	214.682	0.63
	Temperature	215.485	0.62
	Wind	209.45	0.67
Occupancy	Building	213.948	0.69
	CH	215.06	0.47
	CH loss	212.64	0.59
	Road	214.297	0.5
	Water	214.079	0.49

*Note*: Detection variables included temperature (°C), humidity (%), rain (cm), temperature (°C), and wind speed (km/h). Occupancy variables included closest distance to buildings (m), canopy height (“CH”, m), canopy height loss (“CH loss”, m), closest distance to roads (m), and closest distance to water (m).

**FIGURE 2:**
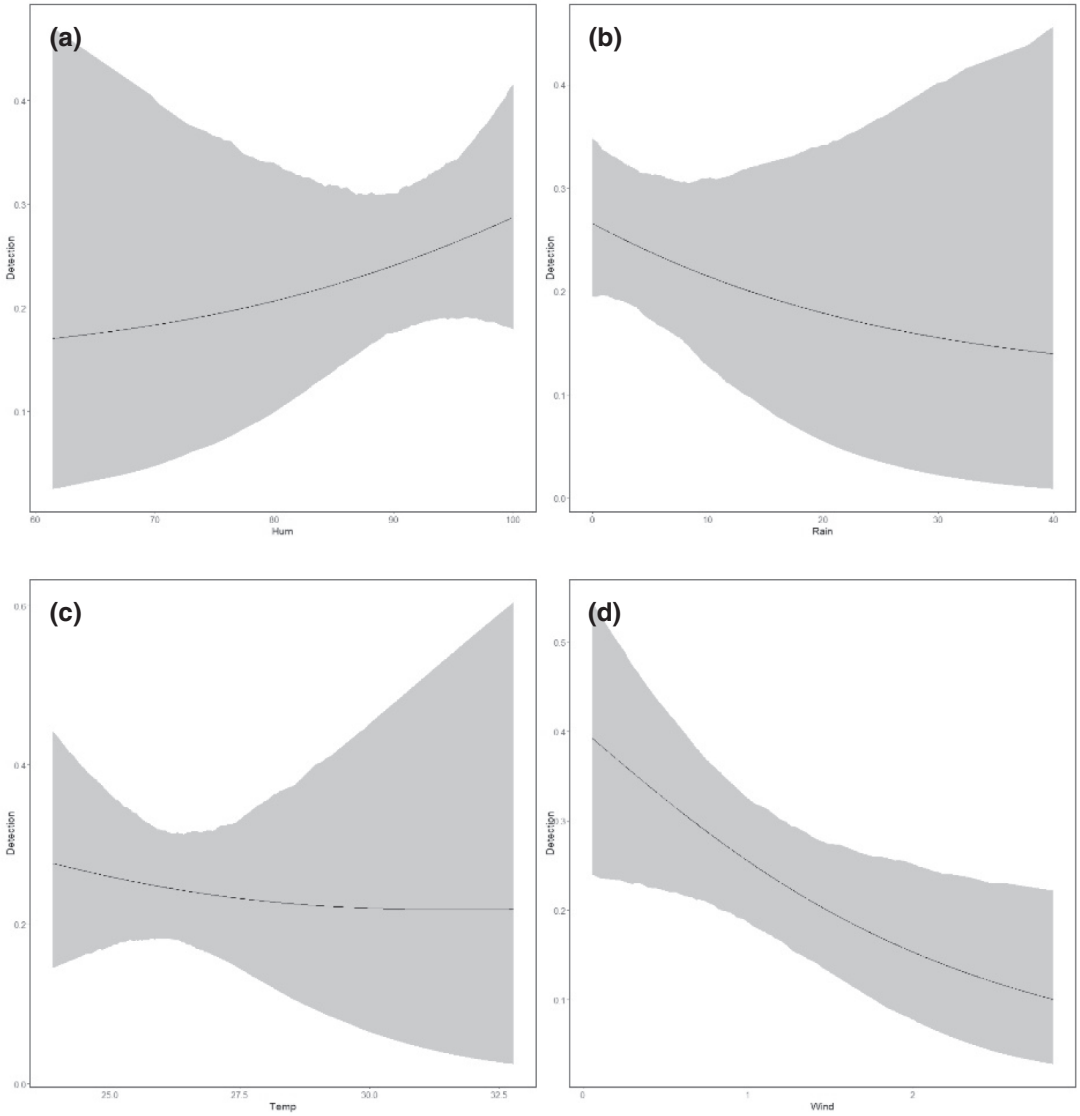
Probability of detecting snakes at Walailak University depending on (a) humidity (“Hum”), (b) rain (“Rain”), (c) temperature (“Temp”), and (d) wind (“wind”) estimated from posterior distribution. The 95% credible intervals are presented in the gray bands.

**FIGURE 3:**
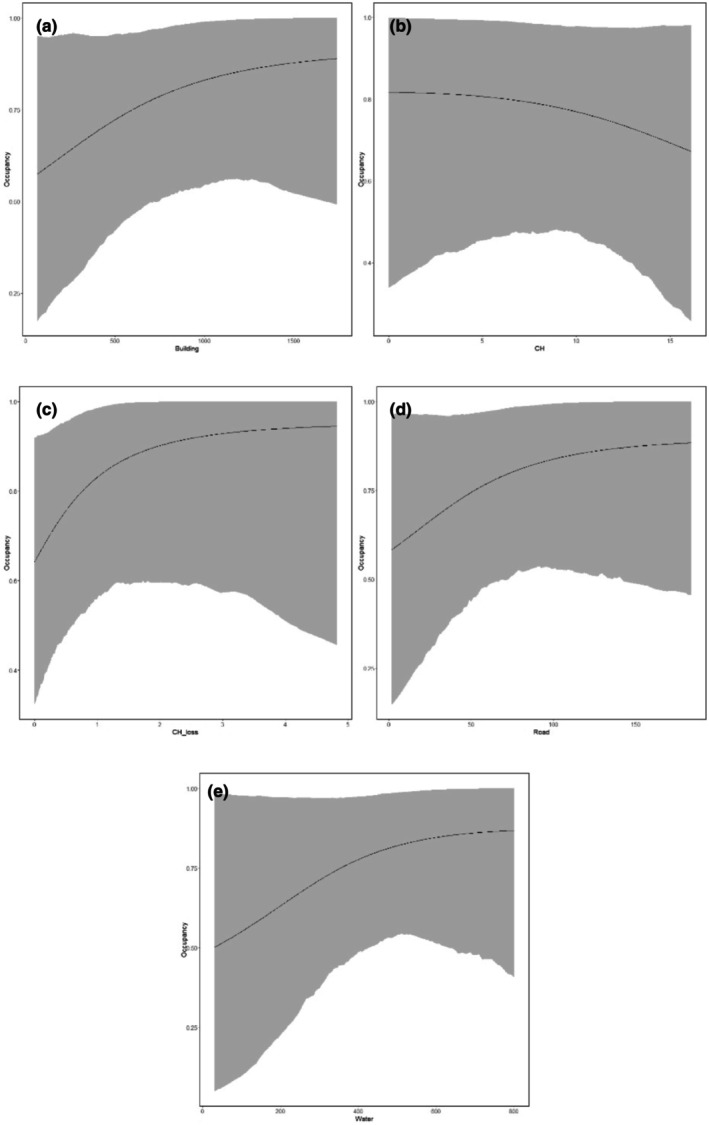
Probability of snakes occupying sites at Walailak University depending on (a) building (“building”), (b) canopy height (“CH”), (c) canopy height loss (“CH_loss”), (d) road (“Road”), and (e) water (“Water”) estimated from posterior distribution. The 95% credible intervals are presented in the gray bands.

The probability of detecting snakes increased with increasing humidity and decreased with increasing amount of rain, temperature, and wind (Figure 2). Site occupancy probability decreased with increased canopy height and increased with increased distance to buildings, increased canopy height loss, increased distance to roads, and increased distance to water (Figure 3). Worth noting is the credible intervals for all models overlapped zero, however, suggesting variable effects to be weak (Table 2). Predicted minimum and maximum detection probabilities varied most with wind speed (range 0.10–0.40) by site, variation of detection of other variables was somewhat lower (range 0.14–0.29).

**TABLE 2:** Summary of single season Bayesian models for estimating snake detection probability and occupancy at Walailak University in 2023.

Model type	Model	Estimate	CRI
Detection	Humidity	0.158	−0.232, 0.561
	Rain	−0.204	−0.638, 0.165
	Temperature	−0.0886	−0.534, 0.340
	Wind	−0.466	−0.871, −0.0876
Occupancy	Building	0.853	−0.549, 2.61
	CH	−0.411	−2.00, 0.875
	CH loss	1.4	−0.358, 4.04
	Road	0.794	−0.7171, 2.79
	Water	0.934	−1.0348, 3.20

*Note*: Detection variables included temperature (“Temp”) and humidity (“Hum”). Detection variables included temperature humidity (%), rain (cm), temperature (°C), and wind speed (km/h). Occupancy variables included closest distance to buildings (m), canopy height (“CH”, m), canopy height loss (“CH loss”, m), closest distance to roads (m), and closest distance to water (m). Credible intervals (“CRI”) are presented alongside parameter estimates (“Estimate”), which may be interpreted similar to that of a confidence interval but instead provide a range of posterior values that includes 95% of the probability.

## 4 DISCUSSION

Few studies have been published investigating snake diversity present on university campuses in Thailand despite retention of natural habitat elements and serving as institutions of higher learning with high potential for inclusion of local and international budding naturalists. Study of general herpetofauna yielded lower numbers of snake species than our work (21 species) at Kasetsart University, Si Racha campus (five species, Duengkae, 2011) and at Suan Sunandha Rajabhat University, Samut Songkhram campus (five species, Chanate & Pattaraporn, 2017) in the central part of Thailand, but was higher at Mahidol University, Kanchanburi campus in the west (24 species, Prasopsin & Aksornneam, 2014). Outside of university campuses, herpetofaunal diversity study has been published for specific protected areas, provinces, and ecoregions within Thailand. Previous published studies exclusively of snake diversity in Thailand is scarce, and prior works have primarily investigated herpetofaunal diversity collectively. Snakes can be uniquely challenging to observe compared to even other squamates (Durso & Seigel, 2015), and findings for snakes can present distinct challenges to human perspectives of these organisms as well as consequences to human health not typically observed for other organisms.

### 4.1 Diversity

Our study presents similar findings of persistence of relatively high reptile diversity in disturbed habitat to other works in Southeast Asia (Wanger et al., 2010) and Thailand, specifically (Crane et al., 2018). Similar to our study, Crane et al. (2018) rarefaction did not reach an asymptote suggesting sampling effort and duration to be a challenge. Extrapolation by Syafiq et al. (2023) for a study investigating snake diversity in Malaysia suggested that an asymptote would not be reached even by doubling their sample size. Snakes as a taxa are difficult to obtain adequate captures to analyze with current statistical methods (Durso et al., 2011; Steen, 2010).

Similar to study of snakes in Malaysia (Syafiq et al., 2023), the family Colubridae was both most frequently encountered in our work as well as being the most species rich taxon. Previous study (Karns et al., 2010) focusing on semiaquatic snakes in Thailand observed quite different findings than our work, with *E. enhydris* the dominant species throughout Thailand and evenness ranging from 0.195 to 0.534–the dominant aquatic species in our study was *H. plumbea* (twice as many observed than *E. enhydris*) and overall snake community evenness (including terrestrial species) was much higher 0.85.

Overall diversity observed in our study (within sites *H* = 0–1.94; *H* = 2.60 collectively) was higher than previous study of sea snakes along the Malay Peninsula captured as bycatch by fishermen (within sites *H*′ = 0.729–1.801; Voris, 2017). Previous study of terrestrial non‐aquatic snakes in Thailand have grouped snakes collectively with lizards and turtles/tortoises (“reptiles”) for diversity analyses, despite the uniqueness of serpents. Furthermore, most studies of terrestrial herpetofauna present only richness (number of species) data, which makes diversity interpretation challenging. Interestingly, in northeast Thailand, Crane et al. (2018) observed average Shannon index values of reptiles to be higher in *Eucalyptus* plantations (*H* = 1.66) and “highly disturbed forest” (*H* = 1.51) than in natural dry dipterocarp forest (*H* = 1.47)–even our individual study sites were heterogeneous (more than 1 habitat type), but direct disturbance classification for snake diversity would be a novel and interesting future study for study of university campuses.

### 4.2 Occupancy and detection probability

Our study sought to encourage scientific best practice; however, this does have implications for comparison and replication of our work. Most significantly, despite utilizing a variety of variables for models, we did not run all combinations of variables in different combinations of models–this “dredging” is usually conducted through selection of variables and combinations of variables resulting in lowest Akaike's Information Criterion (AIC; Akaike, 1974) or Bayesian information criterion (BIC; Schwarz, 1978). Dredged model variables can be collinear and potentially nonintuitive to the real world, run the risk of “collider bias”, and may not be appropriate for inference seeking studies (Stewart et al., 2023). Consequently, interpretation of our study requires recognition that our work utilized relatively few detection and occupancy variables and those variable were selected based off prior observation by the authors. Additional variables almost certainly strongly impact the occupancy and detection of snakes at Walailak, but inclusion of more variables would increase opportunity for collinearity.

While proposed by some as a budget‐friendly and logistical alternative, cessation of surveys after initial detection of a species or taxa can lead to overestimation of detection probability and underestimation of occupancy (Medina‐Romero et al., 2019). Detections of all species of snake was quite limited during quantified surveys (60 snakes) compared to effort and number of sites, which would have resulted in even wider credible intervals than observed for all species collectively, thus, we did not attempt to conduct occupancy and detection analyses for individual species. Lastly, single season occupancy and detection fieldwork is typically conducted within a relatively short period of time, however, we were unable to concentrate adequate number of personal at that temporal scale although our study did still appear to meet model assumptions due to nearly all of our study was conducted within the rain season and personal experience with those sites before and after this current work appeared to suggest that snakes did not violate the presence state occupancy assumption that snakes did not display site colonization or extinction although this could be a concern for future study. While species within our study were fairly distinct in appearance, with limited potential mimicry observed (such as *B. fasciatus* and *L. laoensis*), there were species which could inflict human harm which could be challenging (adequate training) if large numbers of personnel were recruited rapidly for surveys.

Detection probabilities (*p*) below .50 are typically unusual for many organisms, however, it is not irregular for snake studies to report probabilities lower than 0.20 (Durso & Seigel, 2015). Thus, although not ideal for logistics and general statistical analyses, our observed detection probabilities (*p* = .10–.40 for Walailak University as a single site, .14–.29 at sites within the university) are not unusual. Climate can significantly influence both ecology of poikilothermic organisms, including snakes, and human detection (“finding”) of them. Our observation of increased detection probability with humidity was similar to previous study of riparian snakes by Asad et al. (2022) in Sabah, Malaysia Borneo, Malayan pit viper (*C. rhodostoma*) general observations by Daltry et al. (1998) on mainland Malaysia, and green pit viper (*T. macrops*) detection probabilities in Thailand (Barnes et al., 2023). Our observation of decreased detection probability with increased temperatures differed from Barnes et al. (2023) and temperate area studies, however.

Our findings of increased occupancy probability of herpetofauna corresponding with decreased canopy cover and increased canopy cover loss intuitively may initially appear surprising and controversial. However, previous studies in disturbed areas have observed similar findings for herpetofauna. Further south in Songkla and Pattani provinces, Warren‐Thomas et al. (2020) observed positive herpetofauna response to open canopy and open habitat during investigation of rubber plantations and agroforestry, which they attributed to increased detection opportunity by observers (easier to see reptiles in open areas compared to denser forests). We further propose increased thermoregulation opportunity presented by more open habitats to be a proximate cause for increased occupancy probability, and local extinction (immediate surrounding area is residential or agriculture) of species utilizing habitats with more canopy cover as an ultimate cause for observations of increased occupancy probability with decreased canopy cover in our study.

## 5 CONCLUSION

No previous study has approached snake diversity from an occupancy and detection framework within Thailand. Conservation of a green pit viper species was evaluated by Barnes et al. (2023) at a biosphere reserve in Thailand using occupancy and detection, and mammal studies (Chutipong et al., 2014; Ngoprasert & Gale, 2019) regularly employ this methodology in the country also. While occupancy and detection findings are comprehensive, data collected using this method can be more intensive logistically and financially than opportunistic traditional survey methodology utilized for snakes (Durso & Seigel, 2015). Consideration of effort, where and when to survey to avoid bias and maximize findings, and adequate recording of sampling (particularly details within months, rather than general years, and surveyor hours) can be daunting. Understanding of where, when, and how many hours surveys were conducted for previously published herpetofauna surveys in Thailand is challenging, and at best these studies provide biased presence‐only findings. What habitats and under what conditions snakes may be found, providing information of activity by snakes and potential rarity, is difficult if not impossible to derive from these studies, subsequently. Minimum, we suggest better reporting practices for publishing snake diversity, although we promote the occupancy and detection probability framework for the most comprehensive and replicable methodology.

Direct conservation and snakebite management information can be derived from our study. Medically significant species, those which can inflict limb loss, organ failure, and death of humans at Walailak University encountered during our study include banded krait (*B. fasciatus*), Malayan pit viper (*C. rhodostoma*), and monocled cobra (*N. kaouthia*). Worth noting is the red‐necked keelback (*R. siamensis*) has been recorded as inflicting severe bleeding but no mortality in humans. Additionally, despite being a member of the family Elapidae, no severe bites or mortality have been recorded in Thailand for the small spotted coral snake (*C. maculiceps*). The Walailak University campus hospital carries three antivenoms produced by the Thai Red Cross Society‐Malayan pit viper monovalent, monocled cobra polyvalent, and green pit viper polyvalent. We did not detect green pit vipers (genus *Trimeresurus*) on campus nor did we encounter staff and faculty via informal personal discussion who found them locally these species are present within the Nakhon Si Thammarat province, however (Chanhome et al., 2011). Malayan pit vipers were the third most encountered snake species in our study. Although not venomous, the reticulated python (*P. reticulatus*) is the longest species of snake in the world and there have been confirmed records of human predation by the species as well as severe wounds from bites.

Of the 21 species of snakes we detected at Walailak University, five have been deemed threatened with extinction in some capacity locally (WARPA) and internationally (CITES, IUCN). The Indochinese ratsnake (*P. korros*) was the most frequently encountered snake species during the study (36 individuals), with other protected species encountered irregularly (five individuals). We only observed a single case of direct mortality of any snake species, an adult *Fowlea flavipunctatus* which was killed by a local fisherman at one of the ponds, although road mortality of snakes collectively was high. We are proud and cautiously optimistic of the snake diversity currently present at Thailand's largest university campus and encourage follow‐up studies in the future for this budding university with further inclusion of local stakeholders (students, staff, and faculty) at Walailak University and other campuses in the country in herpetofaunal studies.

## AUTHOR CONTRIBUTIONS

**Curt H. Barnes:** Conceptualization (lead); data curation (lead); formal analysis (lead); funding acquisition (lead); investigation (lead); methodology (lead); project administration (lead); writing – original draft (lead); writing – review and editing (lead). **Ungku Zafirah Abdulaziz:** Data curation (equal); investigation (equal); methodology (equal); writing – original draft (supporting); writing – review and editing (supporting). **Arwut Kaenphet:** Conceptualization (equal); investigation (equal); methodology (equal); writing – original draft (supporting); writing – review and editing (supporting). **Chatchai Kanlayanapaphon:** Conceptualization (equal); funding acquisition (equal); methodology (equal); writing – original draft (supporting); writing – review and editing (supporting).

## FUNDING INFORMATION

This research was funded by an individual research grant from Walailak University.

## CONFLICT OF INTEREST STATEMENT

The authors declare that they have no known competing financial interests or personal relationships that could have appeared to influence the work reported in this paper.

## Supporting information


Appendix S1

## Data Availability

Data available on request due to privacy/ethical restrictions and size. Program R code utilized is available in‐text, and can be found at the Open Science Framework repository (diversity, https://osf.io/qgn2p/?view_only=d15e1f7becfa44e5a106111c6069297b; occupancy and detection, https://osf.io/sb37t/?view_only=874f6b5c08e74e4092b483a27d936af6).
