# Supplementary material for: Snake diversity, occupancy, and detection on Thailand's largest university campus
Source: Ecol Evol. 2024 Sep 18;14(9):e70317. doi: 10.1002/ece3.70317 (PMC11410393; doi:10.1002/ece3.70317)

**8- Supplementary Tables**

**Supplementary Table 1.** Snake species detected at Walailak University, Nakhon Si Thammarat province, Thailand from March- October 2023. Included is snake family, species detected, name of snake species in Thai, number of individuals encountered, and method detected (opportunistic, O; road, R; survey, S; trap, T)

| **Family** | **Species** | **Thai name** | **#** | **Method detected** |
| --- | --- | --- | --- | --- |
| **Colubridae** | *Ahaetulla* sp. | งูเขียวหัวจิ้งจก | 10 | O,S,T |
|  | *Chrysopelea ornata* | งูเขียวพระอินทร์ | 4 | O |
|  | *Coelognathus radiatus* | งูทางมะพร้าว | 5 | O |
|  | *Dendrelaphis pictus* | งูสายม่านพระอินทร์ | 5 | T |
|  | *Enhydris enhydris* | งูสายรุ้ง | 12 | O,S |
|  | *Fowlea flavipunctatus* | งูลายสอบ้าน | 9 | O,S |
|  | *Homalopsis mereljcoxi* | งูหัวกะโหลกไทย | 6 | O |
|  | *Hypsiscopus plumbea* | งูปลิง | 34 | T |
|  | *Lycodon laoensis* | งูปล้องฉนวนลาว | 1 | S |
|  | *Oligodon taeniatus* | งูงอดไทย | 12 | O,S |
|  | *Pareas margaritophorus* | งูกินทากจุดขาว | 2 | T |
|  | *Ptyas korros* | งูสิงธรรมดา | 36 | T |
|  | *Rhabdophis siamensis* | งูลายสาบคอแดง | 6 | T |
| **Cylindrophiidae** | *Cylindrophis jodiae* | งูก้นขบลายใหญ่ | 3 | O,S |
| **Elapidae** | *Bungarus fasciatus* | งูสามเหลี่ยม | 1 | S |
|  | *Calliophis maculiceps* | งูปล้องหวายหัวดำ | 1 | R |
|  | *Naja kaouthia* | งูเห่าไทย | 13 | O,S |
| **Pythonidae** | *Python reticulatus* | งูเหลือม | 6 | O |
| **Typhlopidae** | *Argyrophis muelleri* | งูดินใหญ่มลายู | 1 | R |
| **Viperidae** | *Calloselasma rhodostoma* | งูกะปะ | 22 | S, T |
| **Xenopeltidae** | *Xenopeltis unicolor* | งูแสงอาทิตย์ | 6 | S |

**Supplementary Table 2.** Diversity (Shannon index, *H*) and evenness (Pielou’s evenness, *J*) for snake species detected within occupancy study sites (Sites) at Walailak University, Nakhon Si Thammarat province, Thailand in 2023 obtained from opportunistic, road, survey, and trap data. Evenness was not able to be conducted for all sites, indicated by “-“.

| **Site** | ***H*** | ***J*** |
| --- | --- | --- |
| 1 | 0 | 0 |
| 2 | 0 | - |
| 3 | 0.735622 | 0.669592 |
| 4 | 1.94591 | 1 |
| 5 | 1.890363 | 0.909072 |
| 6 | 0 | 0 |
| 7 | 0 | - |
| 8 | 1.028514 | 0.741916 |
| 9 | 0 | 0 |
| 10 | 0.900256 | 0.819448 |
| 11 | 0 | - |
| 12 | 0 | 0 |
| 13 | 0 | 0 |
| 14 | 0 | 0 |
| 15 | 0 | 0 |
| 16 | 0 | 0 |
| 17 | 0 | 0 |
| 18 | 0 | - |
| 19 | 0 | 0 |
| 20 | 0 | - |

**9- Supplementary Figures**

**Supplementary Figure 1**. Rarefaction curve to determine the influence of sample size on species richness, number of individual snakes observed, for snake diversity at Walailak University, Nakhon Si Thammarat province, Thailand in 2023.


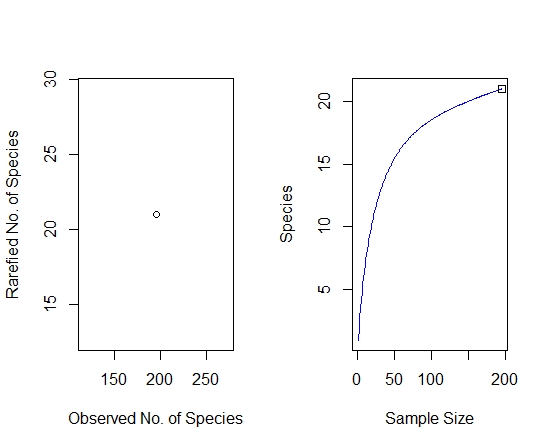


**Supplementary Figure 2**. Rarefaction curve to determine the influence of sample size, number of individual snakes observed, on species richness for snake diversity at 20 sites within Walailak University, Nakhon Si Thammarat province, Thailand in 2023.


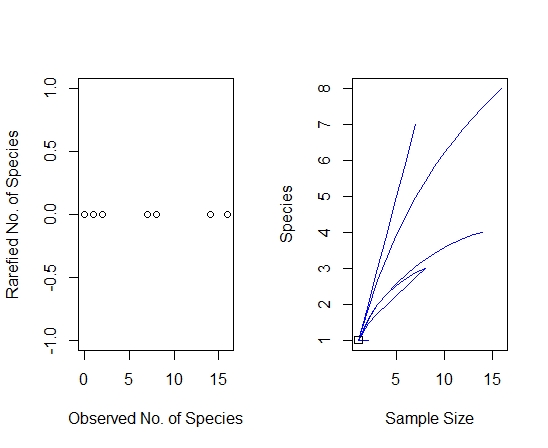


**Supplementary Figure 3**. Accumulation curve to determine the influence of number of sites on species richness for snake diversity at 20 sites within Walailak University, Nakhon Si Thammarat province, Thailand in 2023.


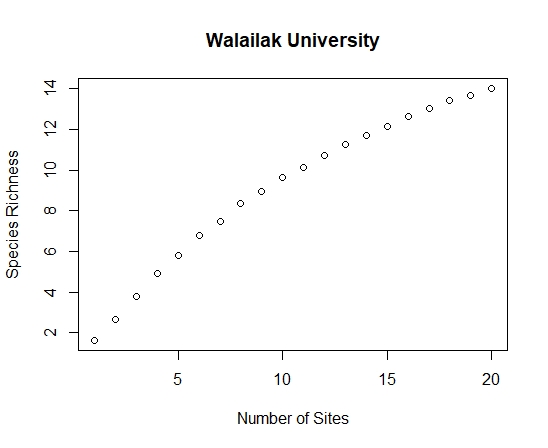

Supplement: Supplementary file 1 — Appendix S1 [file ECE3-14-e70317-s001.docx]
